# Supplementary material for: CD39+PD-1+CD8+ T cells mediate metastatic dormancy in breast cancer
Source: Nat Commun. 2021 Feb 3;12:769. doi: 10.1038/s41467-021-21045-2 (PMC7859213; doi:10.1038/s41467-021-21045-2)
Supplement: Supplementary file 1 — Supplementary Information [file 41467_2021_21045_MOESM1_ESM.pdf]

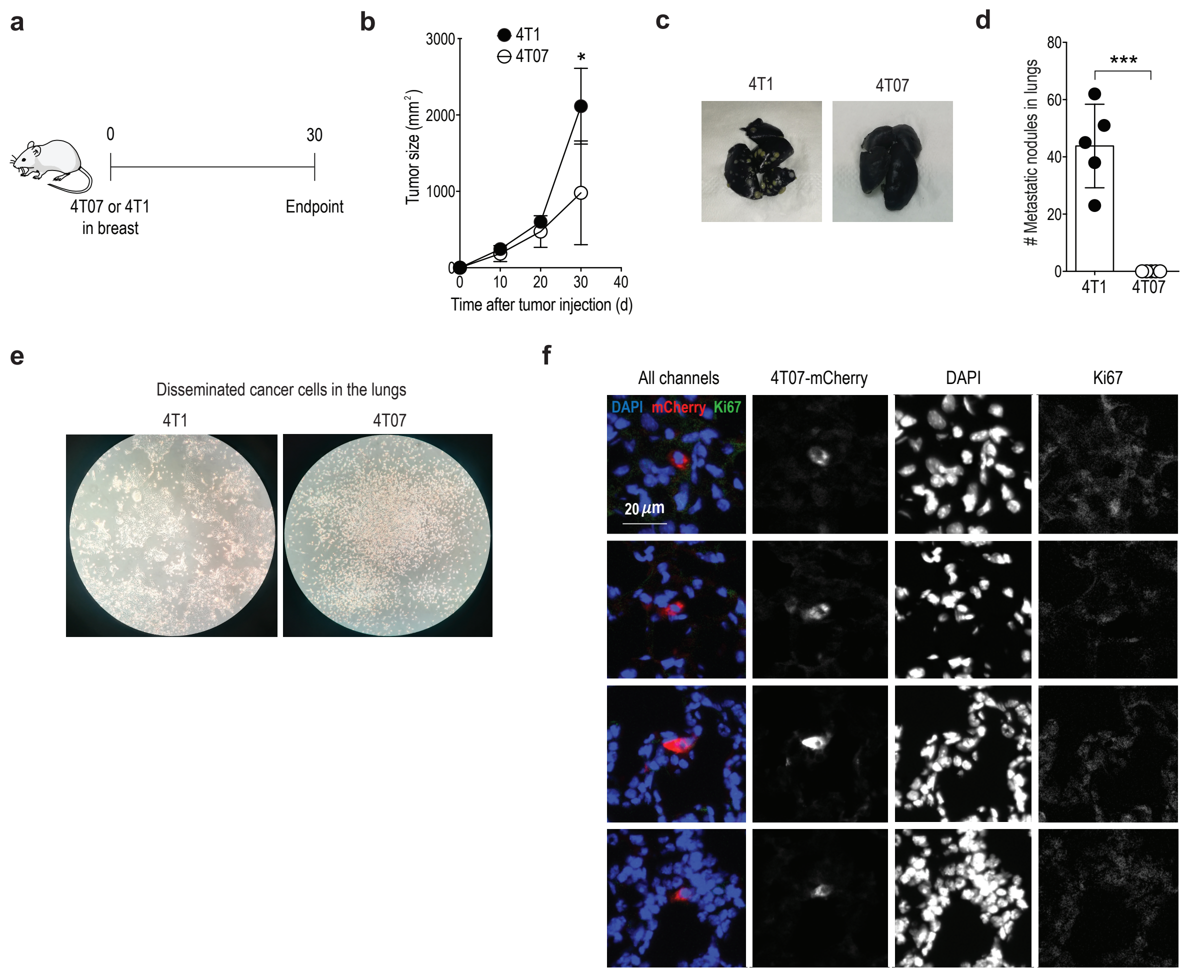

Supplementary Figure 1. **Circulating and disseminated cancer cells in mice with 4T1 or 4T07 orthotopic tumors**. **a** Experimental design. 4T1 or 4T07 cells (10^5) were injected into the mammary fat pad of female BALB/c mice (*n*=5 per group). Analysis was performed 30 d later. **b** Growth curve of primary breast cancer. **c** Representative images of lungs at the endpoint stained with India ink to macroscopically visualize metastatic nodules. **d** Number of metastatic nodules in the lungs. Both groups *n*=5. **p* = 0.0167, ****p* = 0.0002, (2-tailed Student's t-test with Welch's correction). The error bars represent the SD. Results are representative of 3 independent experiments. **e** BALB/c mice were injected with 10^5 4T1 or 4T07 cells in the mammary fat pad and disseminated cancer cells (DCCs) were detected by colony-forming assay in the lungs 30 d later. DCCs were detected in all mice (n=5 per group). Representative images of colonies from cultured lung-digests are shown. **f** 4T07-mCh cells (10^5) were injected into the mammary fat pad of female BALB/c mice. Analysis was performed 35 d later. Disseminated 4T07 cells were found in 5/5 mice and are shown in red, Ki67 in green, DAPI in blue. Representative pictures from a cohort of 5 mice. Scale bar: 20 μm. Results are representative of 3 independent experiments.

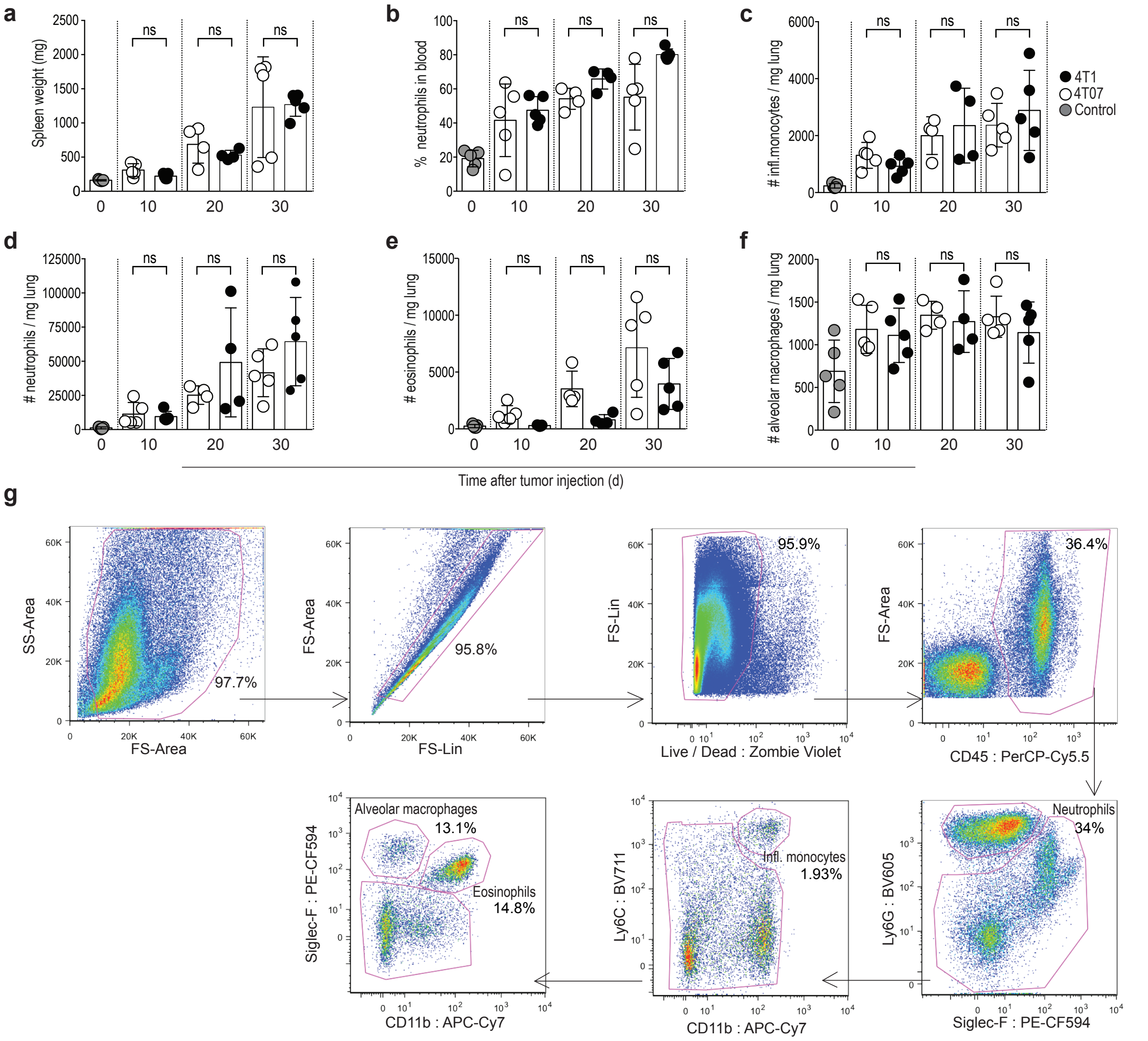

Supplementary Figure 2. **4T07 and 4T1 induce similar systemic inflammation**. Female BALB/c mice were injected with 10^5 4T1 or 4T07 cells in the mammary fat pad. Control mice were injected with PBS. Analysis was performed at the indicated days after cancer cell injection. **a** Spleen weights. **b** Percentage circulating neutrophils of $CD45^+$ cells. **c-f** Total number of cells per mg lung tissue: inflammatory monocytes (**c**), neutrophils (**d**), eosinophils (**e**), alveolar macrophages (**f**). **g** Female BALB/c mice were injected with 10^5 4T1 cells into the mammary fat pad. The plots show a representative gating strategy of lungs processed 20 d after tumor cell injection.

Each symbol represents an individual mouse. Five mice per group. ns = not significant (2-tailed Student's t-test with Welch's correction). The bar represents the mean ± SD.

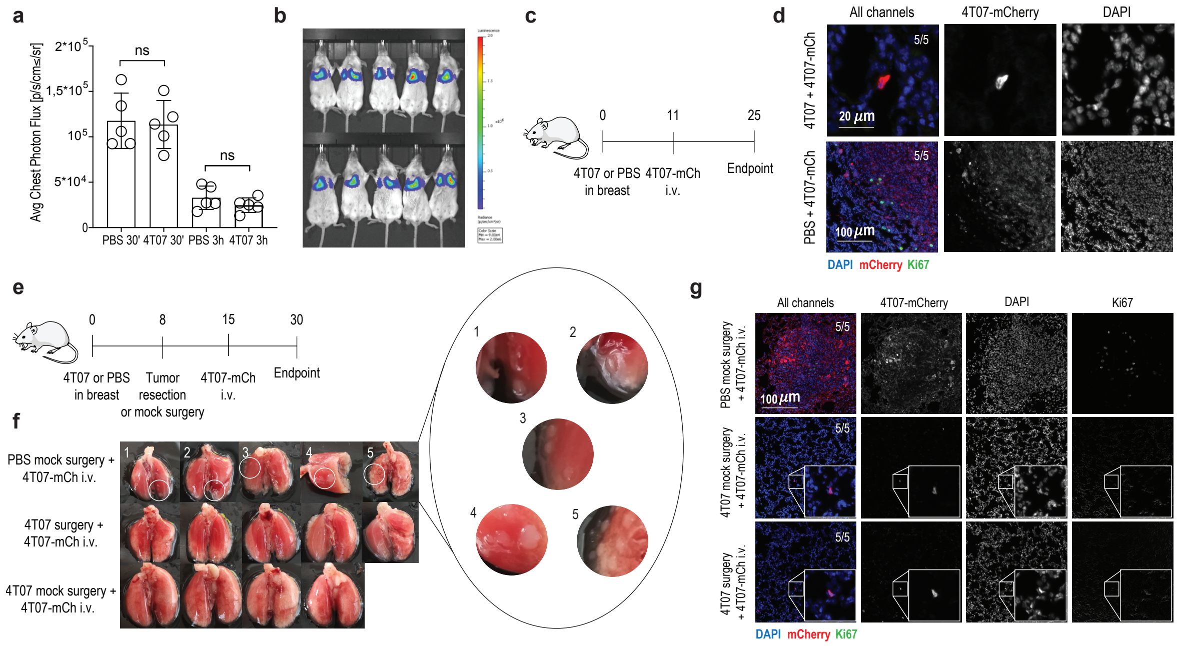

Supplementary Figure 3. **4T07 breast cancer elicits systemic protective immunity. a** 4T07 cells (10^5) or PBS were injected into the mammary fat pad of female BALB/c mice on d 0. On d 11, $3x10^5$ 4T07-LZ cells were injected i.v., and lung seeding was quantified by bioluminescence 30 min and 3 h later. Each group *n*=5. **b** Bioluminescence images. ns = not significant (2-tailed Student's t-test with Welch's correction). Each symbol represents an individual mouse. The bar represents the mean ± SD. Results are representative of 2 independent experiments. **c** Experimental design. 4T07 cells (10^5) or PBS were injected into the mammary fat pad of female BALB/c mice on d 0. On d 11, 10^5 4T07-mCh cells were injected i.v. and analysis of lung metastatic load was performed on d 25. **d** Representative section of the lung at the endpoint. Upper panels: Disseminated 4T07 cells were detected as single, Ki67-negative cells in the lungs of 5 out of 5 mice. Disseminated 4T07 cells are shown in red, DAPI in blue. Lower panels: 4T07-metastatic nodules. Macro-metastasis was detected in 5 out of 5 mice. Scale bar indicates 20 μm. **e** Experimental design. 4T07 cells (10^5) or PBS were injected into the mammary fat pad of female BALB/c mice on d 0. The tumor was resected on d 8; PBS-injected mice and one group of 4T07-injected mice underwent mock resection. On d 15, 10^5 4T07-mCh cells were injected i.v., and analysis of lung metastatic load was performed on d 30. **f** Macroscopic pictures of lungs. Upper row: Metastatic nodules seen in magnifications of the circled areas. Middle and lower row: Absence of macroscopically visible metastatic nodules. **g** Representative example of disseminated 4T07 cells present in the lungs as single, Ki67-negative cells. Disseminated 4T07 cells are shown in red, Ki67 in green, DAPI in blue. Scale bar indicates 20 μm (upper panels) or 100 μm (lower panels).

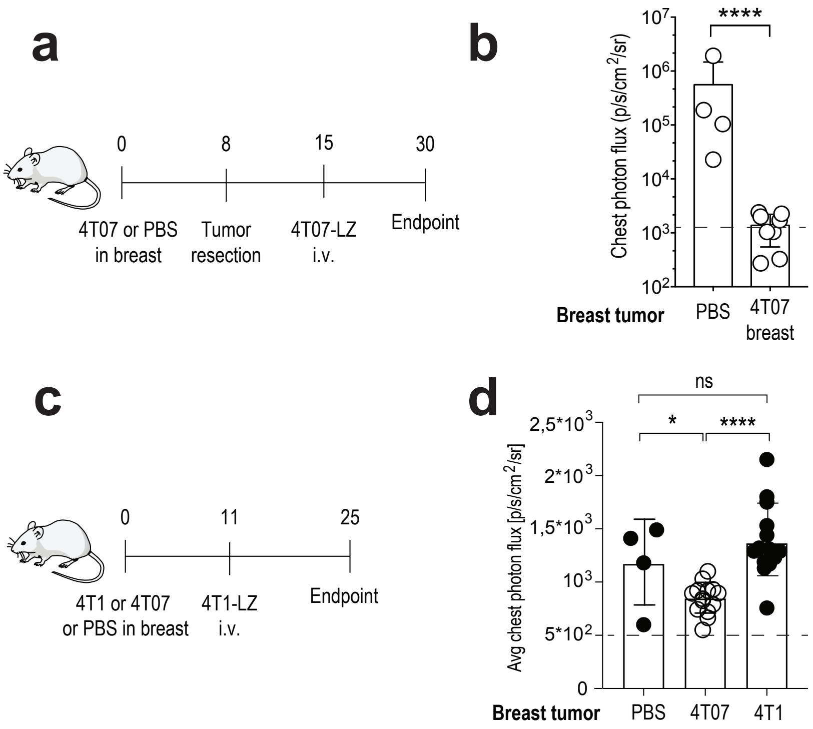

Supplementary Figure 4. **4T07 breast cancer elicits systemic protective immunity. a** Experimental design. 4T07 cells (10^5) or PBS were injected into the mammary fat pad of female BALB/c mice on d 0. The tumor was resected on d 8 (PBS-injected mice underwent mock surgery) and $3x10^5$ 4T07-LZ cells were i.v. injected on d 15. The lung metastatic load was quantified on d 30. **b** Quantification of lung metastatic load by bioluminescence, p < 0,0001, (2-tailed Student's t-test with Welch's correction). Each symbol represents an individual mouse. PBS, *n*=4; 4T07, *n*=9. The bar represents the mean ± SD. Results are representative of 2 independent experiments. **c** Experimental design. 4T1 cells (10^5), 4T07 cells (10^5) or PBS were injected into the mammary fat pad of female BALB/c mice on d 0. On d 11, $3x10^5$ 4T1-LZ cells were injected and analysis of lung metastatic load was performed on d 25. **d** Quantification of lung metastatic load by bioluminescence. PBS-group *n*=4, 4T07 and 4T1 group, *n*=15, p = * 0,018 and **** < 0.0001, ns = not significant (ANOVA with Bonferroni correction). Each symbol represents an individual mouse. The bar represents the mean ± SD.

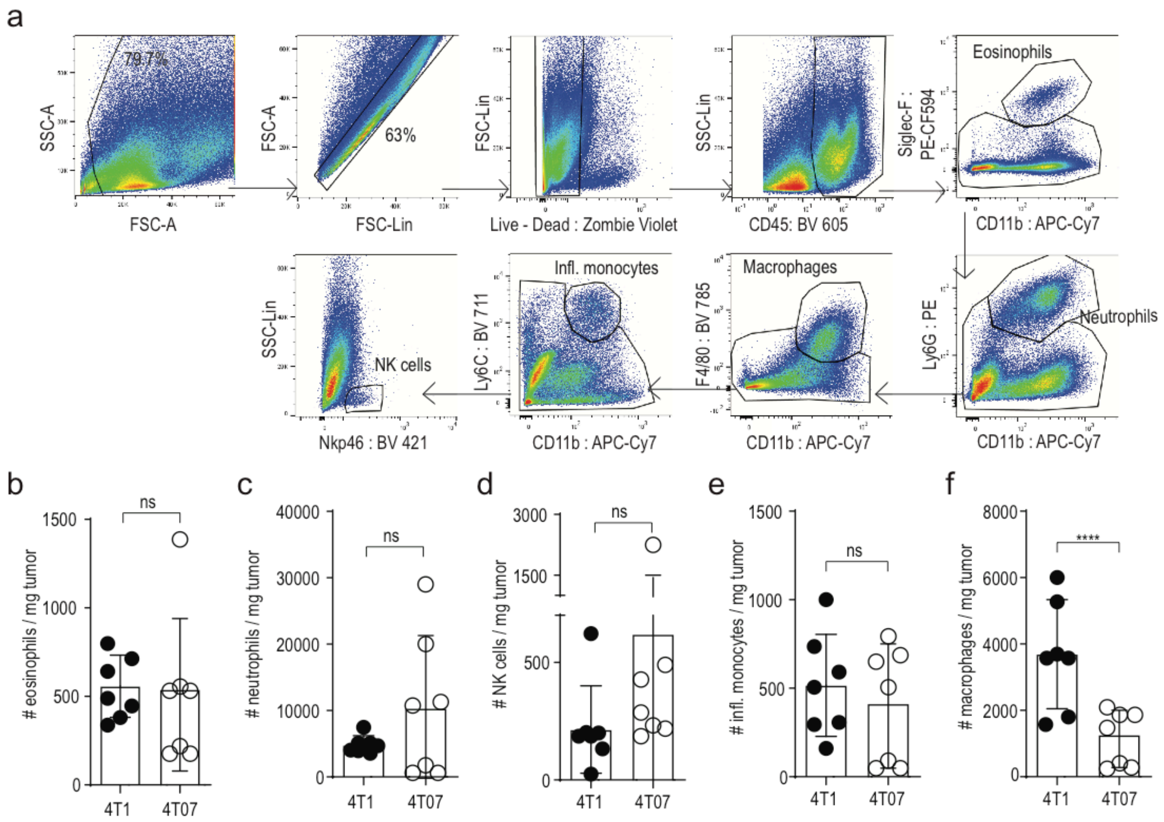

Supplementary Figure 5. **Gating and quantification of tumor-associated leukocytes.** 4T07 cells (10^5) or PBS were injected into the mammary fat pad of female BALB/c mice and tumors were processed for flow cytometry 20 d later. **a** Gating strategy. **b-f** Total number of cells per mg tumor tissue: (**b**) eosinophils, (**c**) neutrophils, (**d**) NK cells, (**e**) inflammatory monocytes, (**f**) macrophages. Each symbol represents an individual mouse. For all plots: 4T1, *n*=7; 4T07, *n*=6. ****$p < 0.0001$ (2-tailed Student's t-test with Welch's correction). The bar represents the mean ± SD.

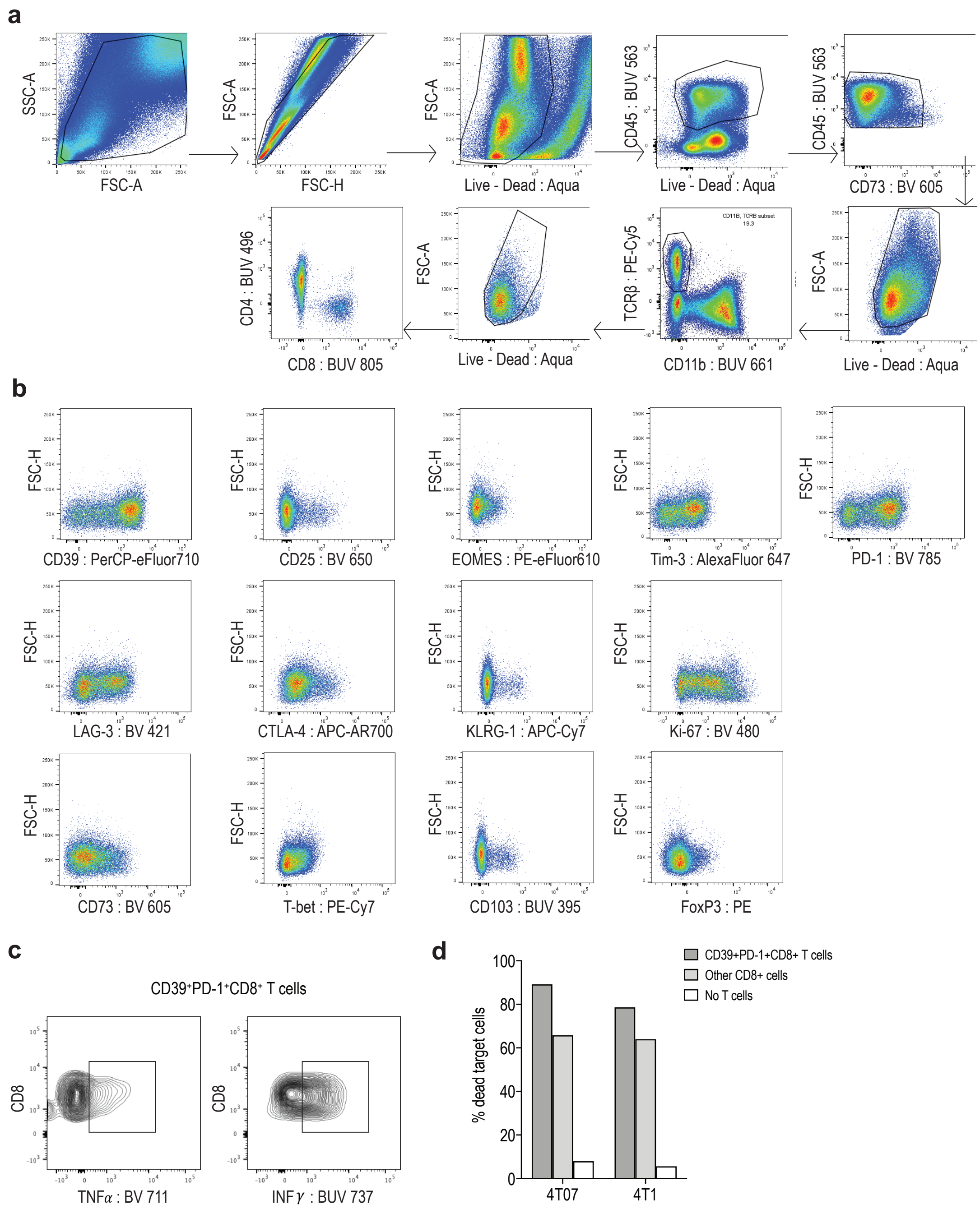

Supplementary Figure 6. **Expression of selected markers by CD39$^+$PD-1$^+$CD8$^+$ T cells.** Female BALB/c mice were injected with 10^5 4T1 or 4T07 cells in the mammary fat pad and primary tumors were analyzed by flow cytometry 20 d later (*n*=4). **a** Gating strategy for Figure 3. **b** Representative examples of expression of selected markers on live, single CD45$^+$ CD44$^+$TCR□$^+$CD11b$^-$CD39$^+$PD-1$^+$CD8$^+$ T cells. **c** Representative examples of IFNγ and TNF□ production by CD45$^+$CD44$^+$TCR□$^+$CD11b$^-$CD39$^+$PD-1$^+$CD8$^+$ T cells sorted from 4T07 tumors after in vitro stimulation with 100 ng/ml phorbol 12-myristate 13-acetate (PMA) plus 1 μg/ml ionomycin for 4 h at 37°C in the presence of GolgiPlug/GolgiStop. **d** CD45$^+$CD44$^+$CD39$^+$PD-1$^+$CD8$^+$ T cells were sorted from 4T07 tumors and incubated for 6 h with 4T07 or 4T1 cancer cells at an E:T ratio of 5. Effectors were cultured overnight with IL-2 plus anti-CD3/CD28-beads before use in the assay. The plot shows the percentage dead target (4T07 or 4T1) cells.

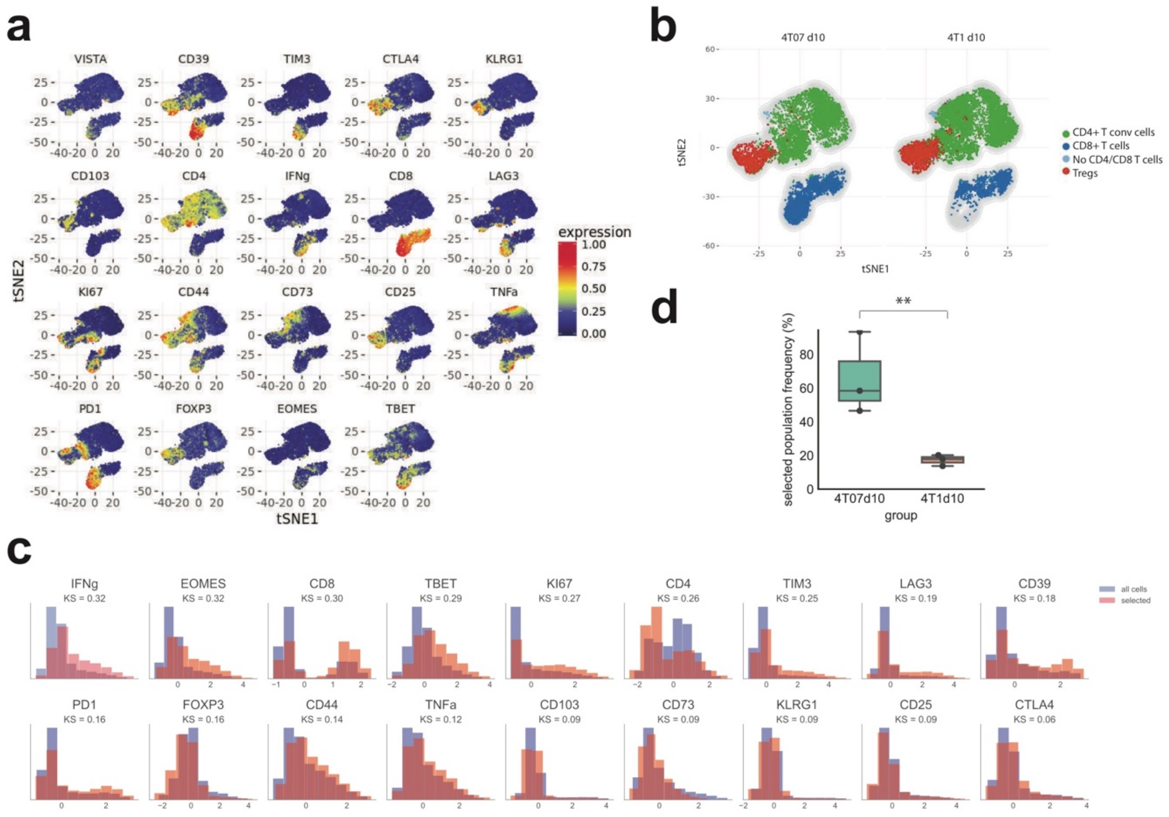

Supplementary Figure 7**. CD39^{+}PD-1^{+}CD8^{+} T cells emerge in dormant breast cancer.** Female BALB/c mice were injected with 10^{5} 4T1 or 4T07 cells in the mammary fat pad and primary tumors were analyzed by flow cytometry 10 d later. **a** t-SNE visualization of markers after gating on single, live, CD45^{+} TCRβ^{+} CD44^{+} cells. **b** t-SNE plots of the main T cell subsets. **c** Scaled histograms of arcsinh-transformed marker expression showing the relative marker distribution of the population identified by CellCnn (red) among all CD8^{+} T cells (blue). KS indicates the Kolmogorov–Smirnov two-sample test between the whole-cell population and the selected cell subsets. **d** Frequency of the population of total T cells identified by CellCnn in both models. Each symbol represents an individual mouse. n=3 per group. $p < 0.001$ (2-tailed Student's t-test with Welch's correction). The bar represents the mean ± SD.

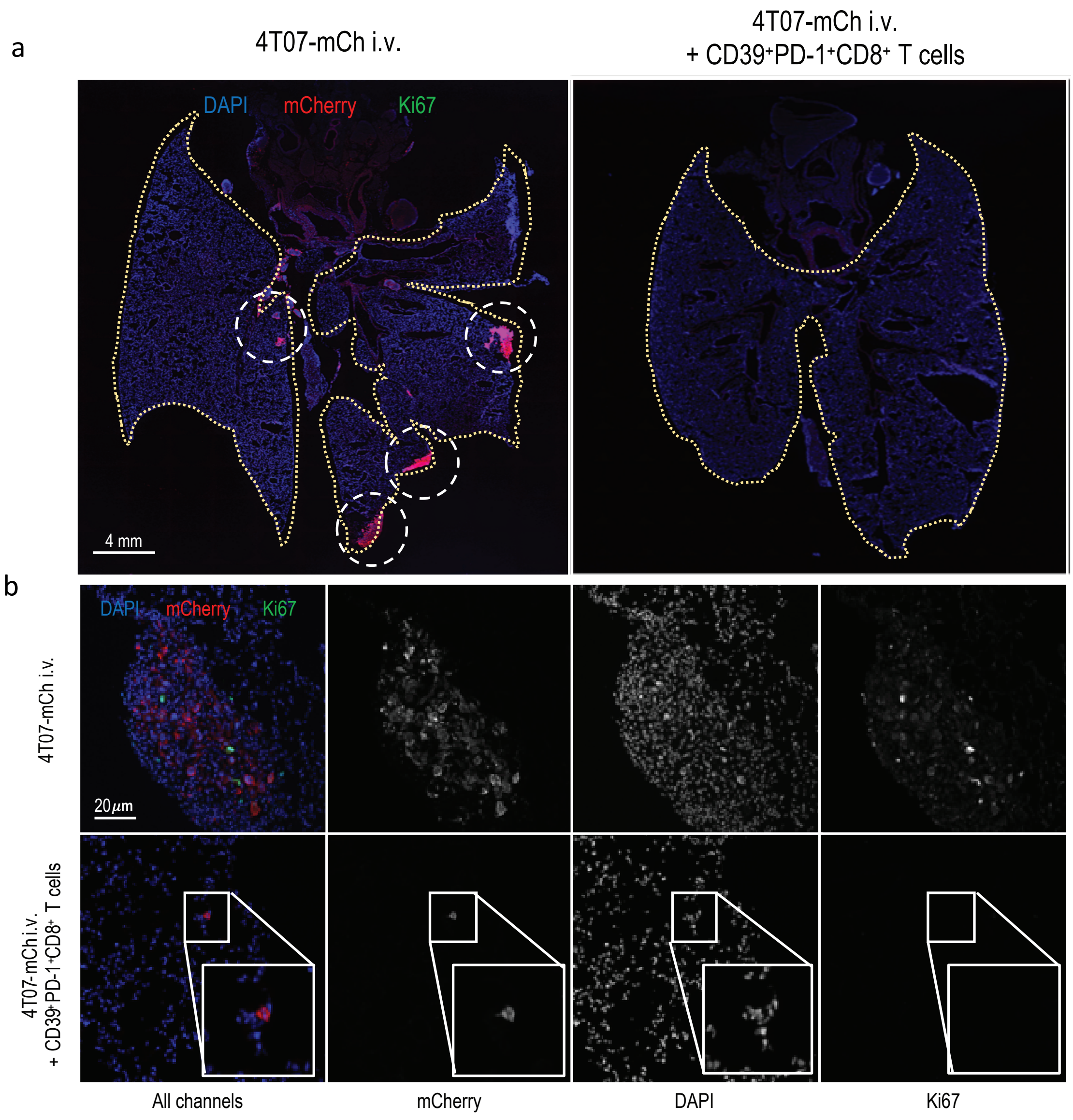

Supplementary Figure 8. **Tumor-associated CD39+PD-1+CD8+ T cells mediate metastatic dormancy**. One-hundred-fifty-thousands sorted CD39+PD-1+CD8+ T cells (or PBS as control) were transferred after injection of $5x10^4$ 4T07-mCh cells into female BALB/c mice. All injections were given intravenously. Lung metastatic load was determined by immunofluorescence 14 d later. Each group consisted of 3 mice. **a** Representative overview images of lungs. The contour of lung tissue is marked by the dotted line. Left panel: Lung of a mouse that received 4T07 + PBS i.v., which resulted in metastatic nodules (indicated by circles). Right panel: Lung of a mouse that received 4T07 + CD39+PD-1+CD8+ T cells i.v.; this lung does not contain metastatic nodules. **b** Representative images of lungs with high magnification. Upper panels: Metastatic nodules in the lungs of mice that received 4T07 + PBS i.v. Lower panels: Single, disseminated dormant 4T07 cells in the lungs of mice that received 4T07 + CD39+PD-1+CD8+ T cells i.v.

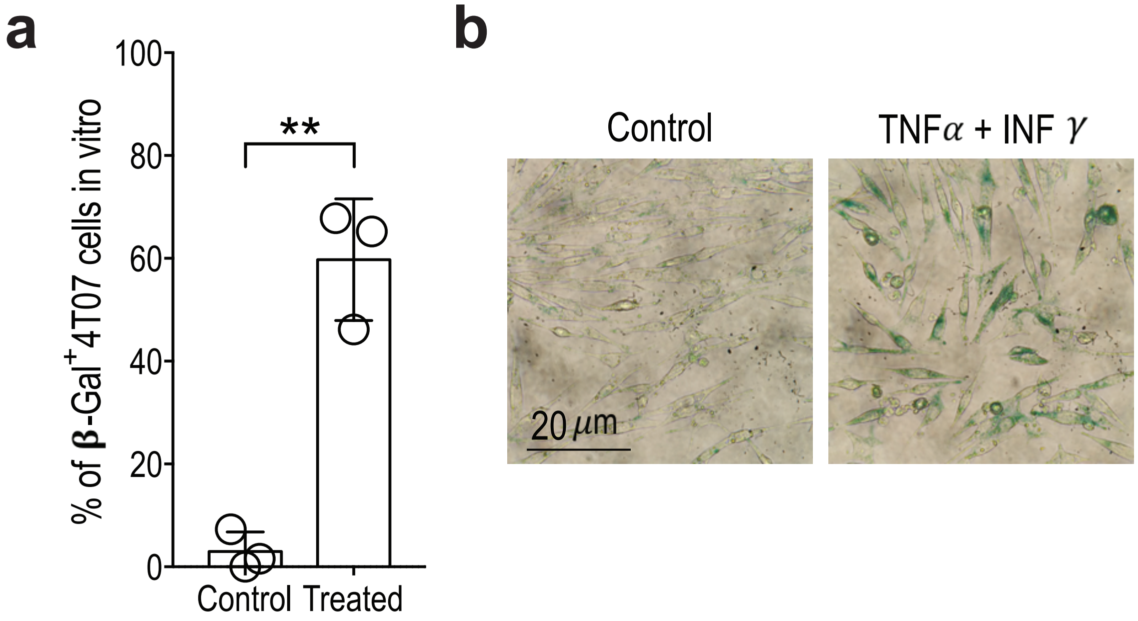

Supplementary Figure 9. **IFNγ and TNFα induce senescence of 4T07 cells.**

Twenty-thousand 4T07 cells were plated in a 6-well plate. Subsequently, 75 ng/ml mouse IFNγ plus 5 ng/ml mouse TNFα were added and cells were incubated for 6 days. **a** Proportion of senescent cells in control and IFNγ plus TNFα -exposed 4T07 cells. Each symbol represents an individual culture, *n*=3, p = 0,0014, (2-tailed Student's t-test with Welch's correction). The bar represents the mean ± SD. Results are representative of 2 independent experiments. **b** Representative examples of staining for SA-β-galactosidase activity (blue) as proxy for senescence. Left panel, medium. Right panel, IFNγ plus TNFα. The experiment was performed twice with similar results.

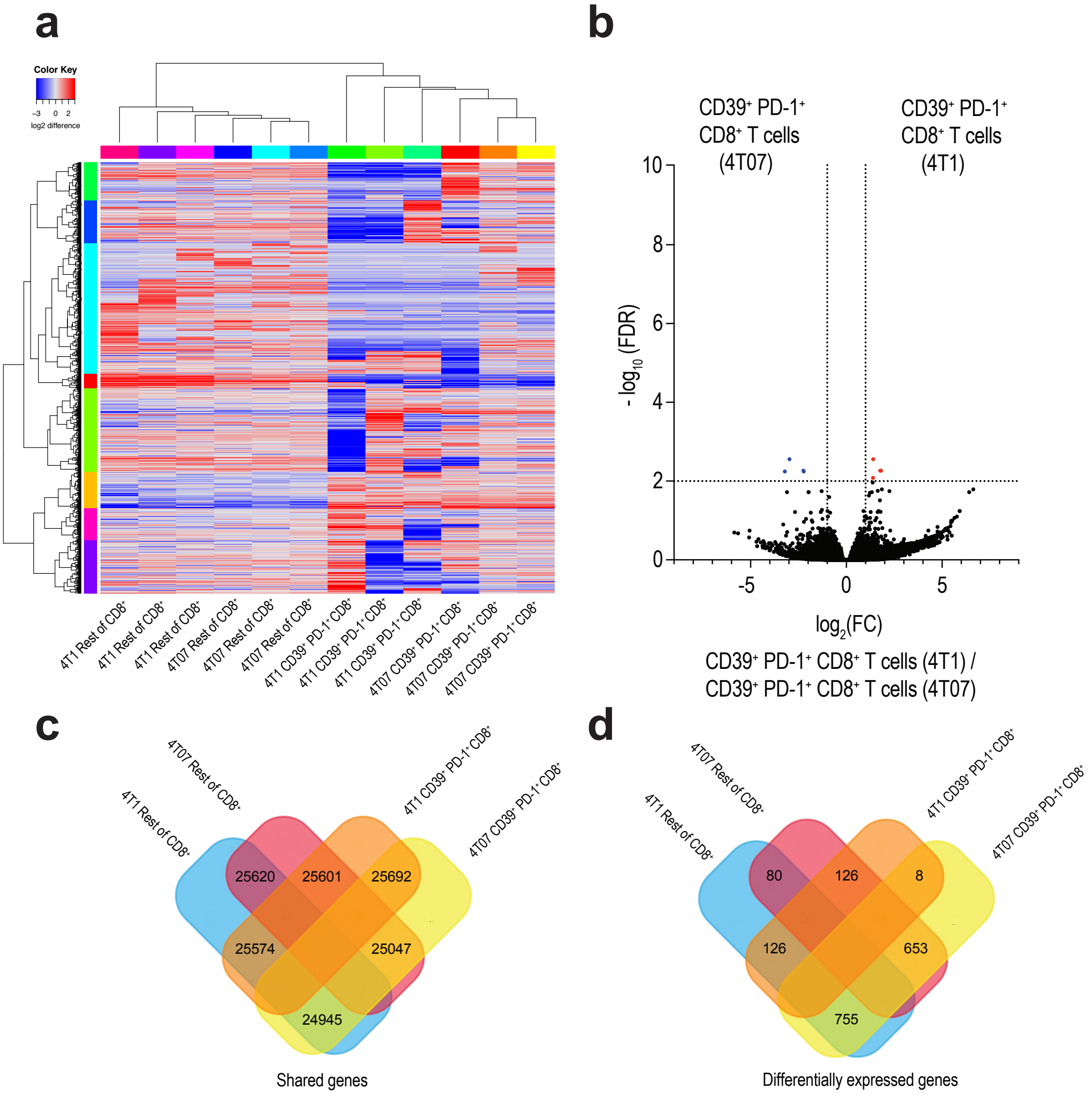

Supplementary Figure 10. **CD39+PD-1+CD8+ T cells in dormant and metastatic tumors have a similar transcription signature. a** Hierarchical clustering of all genes by sorted CD44+CD39+PD-1+CD8+ and CD44+CD8+ T cells. **b** Volcano plot comparing transcripts in CD44+CD39+PD-1+CD8+ T cells sorted from 4T07 breast tumors with those sorted from 4T1 breast tumors. The red symbols represent transcripts that are significantly overexpressed in CD44+CD39+PD-1+CD8+ T cells from 4T07 breast tumors, whereas the blue symbols represent significantly under-expressed transcripts. **c-d** Venn diagram showing (**c**) shared or (**d**) significantly differentially expressed genes in CD44+CD39+PD-1+CD8+ and CD44+CD8+ T cells sorted from 4T1 and 4T07 breast cancers.

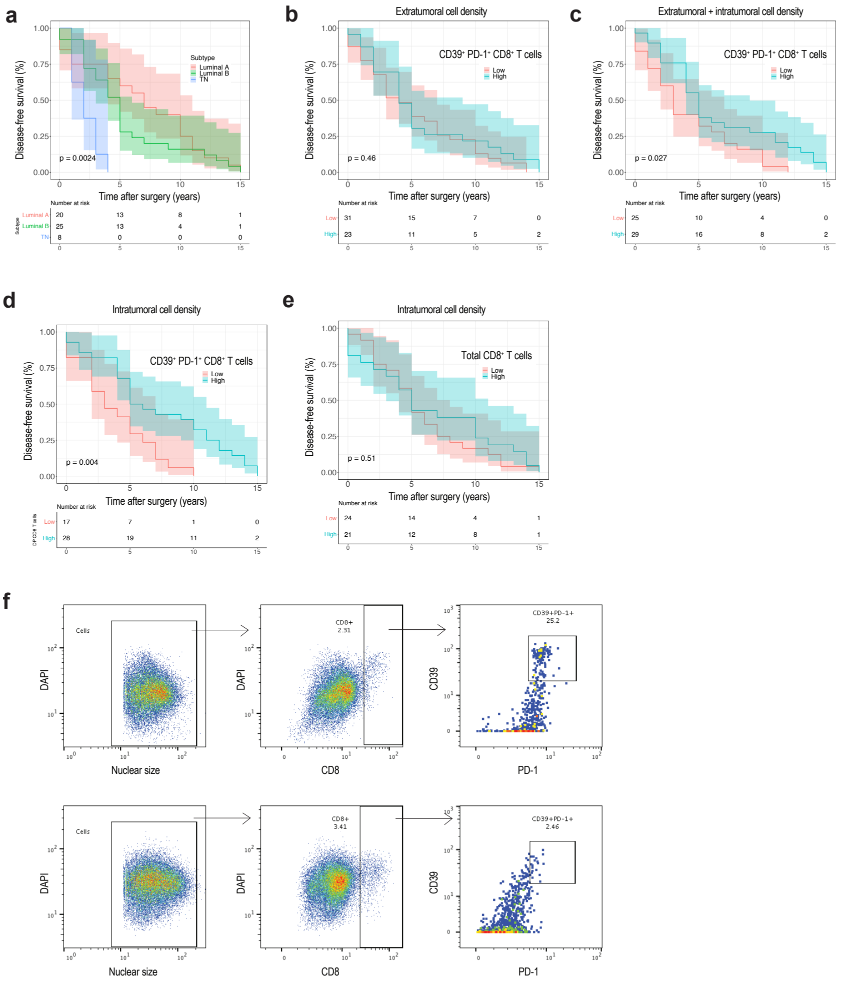

Supplementary Figure 11. **High density of intra-tumoral CD39$^+$PD-1$^+$CD8$^+$ but not total CD8$^+$ T cells correlate with disease-free survival in human luminal A and B breast cancer. a** Disease-free survival of patients with luminal A, luminal B or triple-negative (TN) breast cancer. **b** Disease-free survival of 54 patients with high or low number of extra-tumoral CD39$^+$PD-1$^+$CD8$^+$ T cells. **c** Disease-free survival of 54 patients with high or low number of intra-tumoral + extra-tumoral CD39$^+$PD-1$^+$CD8$^+$ T cells. **d** Disease-free survival of 20 luminal A and 25 luminal B breast cancer patients with high or low number of intra-tumoral CD39$^+$PD-1$^+$CD8$^+$ T cells**. e** Disease-free survival of 20 luminal A and 25 luminal B breast cancer patients with high or low number of intra-tumoral CD8$^+$ T cells**.** ns = not significant. **f** Representative example of a sample with high (upper panels) or low (lower panels) number of intra-tumoral CD39$^+$PD-1$^+$CD8$^+$ T cells. Single-cell parameter files were transformed into .fcs files using R studio and were analyzed using FlowJo software. Values of number of cells/mm^2 were obtained by using the values of total counts obtained from the FlowJo analysis and the measurements of tumor tissue area obtained from the inform analysis. The threshold for separating patients with high and low CD39$^+$PD-1$^+$CD8$^+$ T cell densities was defined using ROC curve analysis. Survival was compared for patients with high and low density of cells to be compared as indicated for the individual graphs by Kaplan-Meier curves. Confidence intervals are indicated as shaded areas surrounding survival curves. Significance was calculated by log-rank test. Patient numbers at risk are displayed for each 15 years of follow-up.

Clinicopathological parameters n=54			
	N°	%	Univariate-Cox *p* value
**Age at initial diagnosis**			0.14
Age < 55	27	50	
Age ≥ 55	27	50	
**Tumor (T) stage**			0.0049
T1	18	33.5	
T2	29	53.5	
T3	4	7	
T4	3	6	
**Nodal (N) stage**			0.64
Negative	23	42.5	
Positive	31	57.5	
**Tumor grade**			0.074
Grade 1	3	5.5	
Grade 2	21	39	
Grade 3	30	55.5	
**ER status**			0.00077
Negative	9	16.5	
Positive	45	83.5	
**PR status**			0.53
Negative	15	28	
Positive	39	72	
**Her2 status**			0.82
Negative	37	66.5	
Positive	17	31.5	
**Molecular subtype**			0.0013
Luminal A	20	37	
Luminal B	25	46	
Her2	1	2	
TN	8	15	
**Density of intratumoral $CD39^{+}PD\text{-}1^{+}CD8^{+}$ T cells**	NA	NA	Multivariate-Cox: Not independent

Supplementary Table 1. **Description of clinicopathological parameters of the human cohort.** Clinicopathological parameters registered for the human cohort. Parameters provided by the University Hospital of Zurich.
