# Supplementary material for: CD39+PD-1+CD8+ T cells mediate metastatic dormancy in breast cancer
Source: Nat Commun. 2021 Feb 3;12:769. doi: 10.1038/s41467-021-21045-2 (PMC7859213; doi:10.1038/s41467-021-21045-2)
Supplement: Supplementary file 3 — Reporting Summary [file 41467_2021_21045_MOESM3_ESM.pdf]

nature research

Corresponding author(s): Maries van den Broek
Last updated by author(s): Dec 15, 2020

Reporting Summary

Nature Research wishes to improve the reproducibility of the work that we publish. This form provides structure for consistency and transparency in reporting. For further information on Nature Research policies, see Authors & Referees and the Editorial Policy Checklist .

Statistics

For all statistical analyses, confirm that the following items are present in the figure legend, table legend, main text, or Methods section.

n/a	Confirmed	
☐	☒	The exact sample size (*n*) for each experimental group/condition, given as a discrete number and unit of measurement
☐	☒	A statement on whether measurements were taken from distinct samples or whether the same sample was measured repeatedly
☐	☒	The statistical test(s) used AND whether they are one- or two-sided<br>*Only common tests should be described solely by name; describe more complex techniques in the Methods section.*
☒	☐	A description of all covariates tested
☒	☐	A description of any assumptions or corrections, such as tests of normality and adjustment for multiple comparisons
☐	☒	A full description of the statistical parameters including central tendency (e.g. means) or other basic estimates (e.g. regression coefficient) AND variation (e.g. standard deviation) or associated estimates of uncertainty (e.g. confidence intervals)
☐	☒	For null hypothesis testing, the test statistic (e.g. *F*, *t*, *r*) with confidence intervals, effect sizes, degrees of freedom and *P* value noted<br>*Give P values as exact values whenever suitable.*
☒	☐	For Bayesian analysis, information on the choice of priors and Markov chain Monte Carlo settings
☒	☐	For hierarchical and complex designs, identification of the appropriate level for tests and full reporting of outcomes
☒	☐	Estimates of effect sizes (e.g. Cohen's *d*, Pearson's *r*), indicating how they were calculated

Our web collection on statistics for biologists contains articles on many of the points above.

Software and code

Policy information about availability of computer code

Data collection	BD FACSDiva software 9.1 - Fortessa LSR II and Symphony Flow cytometer - FACS ARIA III cell sorter (BD Bioscience)<br>Automated multispectral microscopy system Vectra 3.0 (PerkinElmer)<br>IVIS200 imaging system (PerkinElmer)<br>Illumina NovaSeq6000 sequencer (single-end 100 bp)
Data analysis	FlowJo software (version 10, TreeStar Inc)<br>Prism software (GraphPad version 8.0)<br>R-studio (version 1.2.5019) and R-script (version 3.6.1) with standard packages (FlowCore 2.1.0, BioBase 3.12, EdgeR 3.31.3, Survminer 0.4.8, Survival-R 3.2-7, pROC, ROCR). tSNE and FlowSOM algorith mapping live cells were performed as described by Brummelman et al , Nature Protocols 2019.<br>SUSHI software, STAR software (version 2.7.3a), FunRich software (version 3.1.3)<br>GSEA software (version 4.0.3)<br>Inform Software 2.4.8 (PerkinElmer)<br>Cyt3 Matlab software (version 2017b)

For manuscripts utilizing custom algorithms or software that are central to the research but not yet described in published literature, software must be made available to editors/reviewers. We strongly encourage code deposition in a community repository (e.g. GitHub). See the Nature Research guidelines for submitting code & software for further information.

Data

Policy information about availability of data

All manuscripts must include a data availability statement. This statement should provide the following information, where applicable:

- Accession codes, unique identifiers, or web links for publicly available datasets
- A list of figures that have associated raw data
- A description of any restrictions on data availability

The RNA-Seq data are available at https://www.ncbi.nlm.nih.gov/sra/PRJNA609233

Other data are available from the corresponding author upon request.

Field-specific reporting

Please select the one below that is the best fit for your research. If you are not sure, read the appropriate sections before making your selection.

- [x] Life sciences
- [ ] Behavioural & social sciences
- [ ] Ecological, evolutionary & environmental sciences

For a reference copy of the document with all sections, see nature.com/documents/nr-reporting-summary-flat.pdf

Life sciences study design

All studies must disclose on these points even when the disclosure is negative.

Sample size	Mouse experiments: Sample size was calculated so that a sufficient effect size could be detected, using an average standard deviation shown by our previous studies, maintaining a power around 0.80 with alpha 0.05. The calculation above is conducted with R (version 3.6.2) with the function FPower1 in library daewr (ref "Design and Analysis of Experiments with R. John Lawson, 2015). Thus, providing sufficient numbers of mice in each group to yield a 2-sided statistical test with the potential to reject the null-hypothesis.<br>Human samples: The whole cohort of 54 breast cancer patients (including TN, luminal A and luminal subtypes) who underwent tumor resection and were followed up for relapse was used.
Data exclusions	No exclusions. All experimental results were included.
Replication	Biological replicates in each experiment are defined in the figure legends. Most experiments were replicated at least twice with reproducible results. Some studies involving mice were performed only once to limit the use of animals.
Randomization	No randomization was used. Randomization was not necessary because (i) there were no significant differences between groups at the time of intervention, or (ii) the groups were composed of different specimens (for example, 4T1-injected mice / 4T07 injected mice).
Blinding	Investigators were not blinded to the experimental design, due to labeling of mice and cages. Data acquisition using processed samples was done blinded.<br>Human samples were analyzed blinded.

Reporting for specific materials, systems and methods

We require information from authors about some types of materials, experimental systems and methods used in many studies. Here, indicate whether each material, system or method listed is relevant to your study. If you are not sure if a list item applies to your research, read the appropriate section before selecting a response.

Materials & experimental systems

n/a	Involved in the study	
[ ]	[x]	Antibodies
[ ]	[x]	Eukaryotic cell lines
[x]	[ ]	Palaeontology
[ ]	[x]	Animals and other organisms
[ ]	[x]	Human research participants
[x]	[ ]	Clinical data

Methods

n/a	Involved in the study	
[x]	[ ]	ChIP-seq
[ ]	[x]	Flow cytometry
[x]	[ ]	MRI-based neuroimaging

Antibodies

Antibodies used	1. For in vivo depletion:<br>Rat-anti-mouse CD8a (clone YTS169.4), IgG2b, produced in-house

Rat anti-TNP (clone 2A3), IgG2b, BioXCell, cat BE0089
anti-INFy (clone R4-6A2, BioXCell), cat BE0054
anti-TNFa (clone XT3.11, BioXCell), cat BE0058
2. For immunofluorescence
Mouse-anti-human CD39 (clone A1), BioLegend, cat 328202
Rabbit-anti-human PD-1 (clone D4W2J), BioConcept, cat 86163
Mouse-anti-human CD8 (clone RPA-T8), Cell Signaling, cat 561952
Rabbit-anti-human Pan-cytokeratin (polyclonal, H-240), Santa Cruz Biotechnology, cat sc-514686
Rabbit-anti-human EpCAM (clone EPR20532-225), abcam, cat ab223582
Donkey-anti-mouse IgG-HRP (polyclonal) Jackson ImmunoResearch, cat 715-035-151
Donkey-anti-rabbit IgG-HRP (polyclonal Jackson ImmunoResearch, cat 715-035-152)
Goat-anti-mCherry (polyclonal), SICGEN, cat AB8181-200
Rabbit-anti-Ki67 (clone), abcam, ab16667
Donkey-anti-goat IgG-AF594 Jackson ImmunoResearch, cat 705-585-003
Donkey-anti-rabbit IgG-AF488 Jackson ImmunoResearch, cat 711-545-152
3. Flow cytometry
Anti-CD8 in BUV 805, clone 53-6.7, rat IgG2a, BD Pharmingen, cat 612898
Anti-CD11b in BUV 661, clone M1/70, rat IgG2b, BD Pharmingen, cat 612977
Anti-CD45.2 in BUV 653, clone 30-F11, rat IgG2b, BD Pharmingen, cat 550539
Anti-VISTA in AF488, clone MH5A, armenian hamster IgG1, BioLegend, cat 143720
Anti-CD39 in PerCP-eFluor710, clone 24DMS1, rat IgG2b, ebioscience/Thermo Fisher Scientific, cat 46-0391-82
Anti-LAG3 in BV 421, clone C9B7W, rat IgG1, BioLegend, cat 125221
Anti-CD44 in BV 570, clone IM7, rat IgG2b, BioLegend, cat 103037
Anti-CD73 in BV 605, clone TY/11.8, rat IgG1, BioLegend, cat 127215
Anti-CD25 in BV 650, clone PC61, rat IgG1, BioLegend, cat 102038
Anti-PD-1 in BV 785, clone 29F.1A12, rat IgG2a, BioLegend, cat 135225
Anti-TCRb in PE-Cy5, clone H57-597, armenian hamster IgG1, BioLegend, cat 109228
Anti-KLRG1 in APC-Cy7, clone 2F1/KLRG1, armenian hamster IgG1, BioLegend, cat 138426
Anti-TIM3 in AF647, clone B8.2C12, rat IgG1, BioLegend, cat 134006
Anti-CD103 in Biotin, clone 2E7, armenian hamster IgG1, BioLegend, cat 121404
Anti-Streptavidin in BUV 395, BD Pharmingen Cat 612898
Anti-Ki67 in BV 480, clone B56, mouse IgG1, BD Pharmingen, cat 562899
Anti-TNFa in BV711, clone MP6-XT22, rat IgG1, BioLegend, cat 506349
Anti-INFy BUV 737, clone XMG1.2, rat IgG1, BD Pharmingen, cat 564693
Anti-CD4 in BUV 496, clone GK1.5, rat IgG2b, BD Pharmingen, cat 564667
Anti-FOXP3 in PE, clone FJK-16s, rat IgG2a, ebioscience/Thermo Fisher Scientific, cat 12-5773-82
Anti-EOMES in PE-eFluor610, clone Dan11mag, rat IgG2a, ebioscience/Thermo Fisher Scientific, cat 61-4875-82
Anti-T-bet in PE-Cy7, clone eBio4B10, rat IgG1, ebioscience/Thermo Fisher Scientific, cat 25-5825-82
Anti-CTLA4 in APC-AR700, clone UC10-4F10-11, armenian hamster IgG1, BD Pharmingen, cat 564331
Anti-CD24 in FITC, clone M1/69, rat IgG2b, Biolegend, cat 101806
Anti-CD45.2 in APC, clone Ly-5.2, mouse IgG2a, Biolegend, cat 109814

Validation

All antibodies used in our study are commercially available and validated. All antibodies were first confirmed with their specific staining using known positive and negative cells with expected pattern. In house validation was performed for the following antibodies: Anti-CD8 and anti-PD-1 (in healthy human tonsils) and anti-CD39 (in healthy human placenta). All validation samples were kindly provided by the University Hospital of Zurich.

Eukaryotic cell lines

Policy information about cell lines

Cell line source(s)

4T07 cells were a gift from Fred Miller (Karmanos Cancer Institute, Detroit, USA); Variants expressing luciferase, ZsGreen or mCherry were made in-house.
4T1 cells were a gift from Fred Miller (Karmanos Cancer Institute, Detroit, USA); Variants expressing luciferase, ZsGreen or mCherry were made in-house.
Both 4T1 and 4T07 cell lines were produced by Fred Miller ((Aslakson, C. J. & Miller, F. R. Cancer Res 52, 1399–1405. 1992). Thus, both cell lines were obtained from the original source.

Authentication

The original stocks were authenticated. We did not perform further authentication. However, the cell lines were obtained from the original source and behaved as expected in vitro and in vivo and this behavior did not change.

Mycoplasma contamination

Cells were regularly tested negative for Mycoplasma ssp. by PCR analysis in-house. Cells were also tested negative for 18 additional mouse pathogens by PCR (IMPACT II Test, IDEXX Bioanalytics).

Commonly misidentified lines
(See ICLAC register)

No commonly misidentified lines were used.

Animals and other organisms

Policy information about studies involving animals; ARRIVE guidelines recommended for reporting animal research

Laboratory animals	BALB/cJRj and BALB/cAnNRj-Foxn1nu/nu were purchased from Janvier labs (Roubaix, FR). NOD.Cg-PrkdcscidIl2rgtm1Wjl/SzJ mice were obtained from the Jackson Laboratory. All mice were females, 8-10 weeks old at the moment of start of the experiments. All the animals were housed in conditions of 22°C and 45-65% humidity, with 12h light cicle.
Wild animals	This study did not involve wild animals.
Field-collected samples	This study did not involve field-collected samples.
Ethics oversight	All experiments were performed in accordance with the Swiss federal and cantonal regulations on animal protection and were approved by The Cantonal Veterinary Office Zurich, Switzerland (156/2018).

Note that full information on the approval of the study protocol must also be provided in the manuscript.

Human research participants

Policy information about studies involving human research participants

Population characteristics	Tumor tissues from 54 patients with breast cancer (including TN, luminal A and luminal subtypes) were collected at the University Hospital Zurich, Switzerland. Further characteristics of the cohort are provided in Table 1 of the manuscript.
Recruitment	The cohort was established with the intention to eventually compare the immune infiltrate among primary breast cancer tissue and intrapatient matched distant metastatic sites. To this end we searched breast cancer patients suffering from either invasive-ductal or invasive-lobular breast cancer with hematogenous metastases in the archives of the Department of Pathology and Molecular Pathology, University Hospital Zurich. Our cohort is therefore biased for patients with advanced metastatic disease and differs from an average breast cancer cohort. While we cannot formally exclude any bias by this cohort selection we are not aware of any studies systematically comparing the tumor immune microenvironmnent in primary breast cancer between metastatic and non-metastatic cases.
Ethics oversight	Donors provided written, informed consent to tissue collection, analysis and data publication according to the Declaration of Helsinki. Law abidance was reviewed and approved by the ethics commission of the Canton Zurich (BASEC-Nr. 2018-02282 and KEK-ZH-2013-0584). Samples were numerically coded to protect donors' rights to confidentiality and privacy.

Note that full information on the approval of the study protocol must also be provided in the manuscript.

Flow Cytometry

Plots

Confirm that:

- [x] The axis labels state the marker and fluorochrome used (e.g. CD4-FITC).
- [x] The axis scales are clearly visible. Include numbers along axes only for bottom left plot of group (a 'group' is an analysis of identical markers).
- [x] All plots are contour plots with outliers or pseudocolor plots.
- [x] A numerical value for number of cells or percentage (with statistics) is provided.

Methodology

Sample preparation	Animals were euthanized by isoflurane overdose. The right heart ventricle was perfused with 10 ml PBS to eliminate the blood from the lung vessels. Primary tumors and lungs were collected in DMEM, cut into small pieces and digested for 45 minutes at 37°C in DMEM containing 1 mg/ml collagenase IV and 2.6 µg/ml DNase I (both Sigma) on a rotating device. Samples were washed with PBS by centrifugation for 5 minutes at 350 g, the pellet was suspended in PBS and filtered through a 70-µm filter (BD Biosciences) to obtain a single cell suspension. For lymphocyte analysis, cells were further purified by centrifugation over a Percoll gradient (GE Healthcare, 17-0891-01, Sigma Aldrich).<br>Single cells were stained according to standard protocols.
Instrument	Fortessa LSR II and Symphony Flow cytometer - FACS ARIA III cell sorter (BD Bioscience)
Software	BD FACSDiva software, FlowJo software (version 10, TreeStar Inc), R-Studio
Cell population abundance	Populations are described by appropriate markers, which is illustrated in gating strategies. The abundance of populations is expressed as frequency or absolute number as specified in the legends.
Gating strategy	Conventional flowcytometry:<br>FMOs were used for determining the threshold between positive and negative cells.

- [x] Tick this box to confirm that a figure exemplifying the gating strategy is provided in the Supplementary Information.
